# Pulmonary Responses of Sprague-Dawley Rats in Single Inhalation Exposure to Graphene Oxide Nanomaterials

**DOI:** 10.1155/2015/376756

**Published:** 2015-07-30

**Authors:** Sung Gu Han, Jin Kwon Kim, Jae Hoon Shin, Joo Hwan Hwang, Jong Seong Lee, Tae-Gyu Kim, Ji Hyun Lee, Gun Ho Lee, Keun Soo Kim, Heon Sang Lee, Nam Woong Song, Kangho Ahn, Il Je Yu

**Affiliations:** ^1^Toxicology Laboratory, College of Animal Bioscience and Technology, Konkuk University, Seoul 143-701, Republic of Korea; ^2^Institute of Nano Products Safety Research, Hoseo University, Asan 336-795, Republic of Korea; ^3^Occupational Lung Diseases Institute, KCOMWEL, Incheon 403-711, Republic of Korea; ^4^Department of Mechanical Engineering, Hanyang University, Ansan 426-791, Republic of Korea; ^5^Department of Chemical Engineering, Dong-a University, Busan 602-714, Republic of Korea; ^6^Center for Nanosafety Metrology, Korea Research Institute of Standards and Science, Daejeon 305-340, Republic of Korea

## Abstract

Graphene is receiving increased attention due to its potential widespread applications in future. However, the health effects of graphene have not yet been well studied. Therefore, this study examined the pulmonary effects of graphene oxide using male Sprague-Dawley rats and a single 6-hour nose-only inhalation technique. Following the exposure, the rats were allowed to recover for 1 day, 7 days, or 14 days. A total of three groups were compared: control (fresh air), low concentration (0.46 ± 0.06 mg/m^3^), and high concentration (3.76 ± 0.24 mg/m^3^). The exposure to graphene oxide did not induce significant changes in the body weights, organ weights, and food consumption during the 14 days of recovery time. The microalbumin and lactate dehydrogenase levels in the bronchoalveolar lavage (BAL) fluid were not significantly changed due to the exposure. Similarly, total cell count, macrophages, polymorphonuclear leukocytes, and lymphocytes were not significantly altered in the BAL fluid. Plus, the histopathological examination of the rat lungs only showed an uptake of graphene oxide in the alveolar macrophages of the high-concentration group. Therefore, these results demonstrate that the single inhalation exposure to graphene oxide induce minimal toxic responses in rat lungs at the concentrations and time points used in the present study.

## 1. Introduction

Graphene is a two-dimensional graphitic carbon nanomaterial with unique physical and chemical properties that enable a wide variety of applications [1]. Plus, graphene oxide is a graphene nanomaterial that contains oxygen functional groups, such as carboxylates, epoxides, and hydroxyls [2, 3]. Since its first introduction in 2004, the application of graphene has been extensively studied across a variety of areas, including electronics, energy, batteries, flexible displays, sensors, biomedicine, and biotechnology [4]. However, the rapid development and widespread application mean that the potential for human exposure to such nanomaterials is also increasing, which has raised concerns about the safety of nanomaterials in occupational and environmental settings [5].

As a result, several toxicological studies have recently been performed to determine the toxicity of graphene nanomaterials. For example, graphene nanoplatelets were found to accumulate in mouse lungs for up to 28 days after a single intratracheal instillation at a dose of 2.5 or 5.0 mg/kg [4]. Plus, at these concentrations, inflammatory cytokines, such as tumor necrosis factor-*α* (TNF-*α*), tumor growth factor-*β* (TGF-*β*), and interleukin-1*β* (IL-1*β*) and IL-6, were significantly elevated in the bronchoalveolar lavage (BAL) fluid during the recovery period of 28 days. In another study, graphene exposure increased the polymorphonuclear neutrophil (PMN) levels in the BAL fluid from male Wistar rats following exposure to graphene at target concentrations of 2.5 or 10.0 mg/m^3^ for 6 hours per day for 5 consecutive days with 7 or 28 days of recovery [5]. In addition, the lung tissue levels of IL-1*β* were also increased at day 7 in a dose-dependent manner, while graphene-loaded alveolar macrophages were observed in the lungs of all the treated animals in a concentration-dependent manner. Meanwhile, when using a pharyngeal aspiration technique with female C57BL/6 mice, graphene nanoplatelets (50 *μ*g) were found to increase the pulmonary PMN number and proinflammatory cytokines, such as monocyte chemotactic protein-1 (MCP-1), macrophage inflammatory protein-1*α* (MIP-1*α*), and IL-1*β*, in the BAL fluid [6]. A histological examination of the mouse lungs treated with graphene nanoplatelets also showed granulomatous lesions in the bronchiole lumen and near the alveolar region. Finally, the toxic effects of graphene nanomaterials have also been investigated in cultured cells, where several toxic responses, such as increased proinflammatory cytokines and decreased cell viability, have been reported following graphene exposure [4, 6].

Thus, according to these findings obtained from* in vivo* and* in vitro* studies, the inhalation of graphene nanomaterials would be expected to exert toxicity in humans, as in experimental animals. However, since only a small amount of data with limited experimental designs has so far been reported, more data obtained from a variety of experimental settings needs to be accumulated. We also hypothesized that relatively lower concentration of graphene exposure would represent more valuable outcome in order to translate into a human exposure scenario. Also, the selected concentrations were based on the previous experimental setting employed in inhalation toxicity studies for graphene nanomaterials [5]. In addition, no publication has been reported regarding pulmonary effects of graphene oxide nanomaterials following acute inhalation exposure of animals. Therefore, the current study evaluated the toxic effects of graphene oxide nanomaterials in the lungs of Sprague-Dawley rats. The rats were exposed to two different concentrations of graphene oxide (target concentrations: 0.3 mg/m^3^ or 3 mg/m^3^) for 6 hours using a nose-only inhalation chamber and then allowed to recover for 1 day, 7 days, or 14 days. The results showed that the graphene oxide nanomaterials produced minimal toxic effects in the male rats for the experimental settings used in this study.

## 2. Materials and Methods

### 2.1. Characterization of Graphene Nanopowder

The graphene oxide nanomaterials were kindly provided by Dr. Heon Sang Lee of Dong-A University (Busan, Korea). A transmission electron microscope equipped with an energy-dispersive X-ray analyzer (TEM-EDX) was used to measure the graphene oxide nanomaterials based on National Institute for Occupational Safety and Health (NIOSH) analytical method 7402 [7]. The graphene oxide was mounted on a TEM grid (copper grid) and visualized under a field emission-transmission electron microscope (FE-TEM, JEM2100F, JEOL, Japan). The nanomaterials were measured at a magnification of 100,000 and accelerating voltage of 200 kV. The graphene oxide elements were also analyzed using an energy-dispersive X-ray spectrometer (EDS, TM200, Oxford, UK). An X-ray diffraction (XRD) analysis was performed directly on the hybrid samples using Rigaku-Ultima IV (40 kV, 40 mA, Tokyo, Japan) with Cu irradiation at a scanning rate of 0.02/s in the range of 2–40 degrees. In addition, the average flake size of the graphene oxide in a solution was measured using dynamic light scattering (DLS).

### 2.2. Aerosol Generation

Male Sprague-Dawley (SD) rats were exposed to the graphene oxide nanomaterials using a nose-only exposure system (NITC system, HCT Co., Ltd., Incheon, Korea). The nose-only exposure system provided reliable exposure condition with accuracy and consistency. Thus, this exposure system was extensively used for inhalation of carbon-based nanomaterials including graphene in our laboratory and by others [8–10]. The graphene oxide was generated using an atomizer, and purified air was used as the carrier gas. The gas flow was maintained at 16 liters per minute (L/min) using a mass flow controller (MFC, AERA, FC-7810CD-4V, Japan), and the flow rate to each nose port was 1 L/min. The AC power supply was maintained at 99.56 ± 0.07 V (mean ± SE). The target concentrations of the generated graphene oxide were 0.3 mg/m^3^ and 3 mg/m^3^ for the low- and high-concentration chamber, respectively.

### 2.3. Animals and Conditions

The six-week-old male specific-pathogen free SD rats were purchased from OrientBio (Seongnam, Korea) and acclimated for 2 weeks before initiating the inhalation exposure. During the acclimation and inhalation exposure, the rats were housed in polycarbonate cages (maximum of 3 rats per cage) installed in individually ventilated cage racks. The rats were kept under a controlled temperature (21.8 ± 0.17°C) and humidity (49.16 ± 2.37%) and a 12 h light/dark cycle. The rats were fed a rodent diet (Woojung BSC, Suwon, Korea) and filtered water* ad libitum*. During the acclimation period, the animals were trained to adapt to the nose-only inhalation chamber. The rats were divided into 3 groups: control (unexposed, *n* = 12), low-concentration group (*n* = 12), and high-concentration group (*n* = 12). The low- and high-concentration groups were exposed to the graphene oxide for a single period of 6 hours, while the control group received filtered fresh air. The animals were examined daily for any evidence of exposure-related toxic responses. The body weights were measured at the time of purchase, at the time of grouping, after the 6 h inhalation exposure, and before necropsy. The food consumption (g/rat/day) was measured once a week. After the single 6 h inhalation exposure to graphene oxide, the rats were allowed to recover for 1 day, 7 days, or 14 days (*n* = 4 per treatment group for each time period) to investigate the toxic responses. At sacrifice, gross observations of the organs were recorded, and the testes, kidneys, spleen, liver, lungs, and brain were all carefully removed and weighed. All the animal protocols were approved by Hanyang University Institutional Animal Care and Use Committee.

### 2.4. Monitoring of Inhalation Chamber and Analysis of Graphene Oxide Nanopowder

The concentrations of graphene oxide in the chambers were measured using polycarbon filters connected to a MAS Escort ELF sampling pump (MSA, Pittsburgh, PA, USA) at a flow rate of 1.0 L/min. The weight difference of the polycarbon filter before and after sampling was calculated. The size distribution of the graphene oxide was then measured directly using a scanning nanoparticle spectrometer (SNPS, HCT Co., Ltd., Korea) connected to a condensation particle counter (CPC, model 3022A, TSI Inc., Shoreview, MN, USA) and dust monitor (Model 1.1.09, Grimm Technologies Inc., Douglasville, GA, USA). The volume of sheath air and polydispersed aerosol air used in the SNPS and CPC was 15 and 1.5 L/min, respectively.

### 2.5. Bronchoalveolar Lavage (BAL) Cell Evaluation

At sacrifice, the right lungs were 4 times with 3 mL aliguots of cold 0.9% NaCl solution (PBS, pH 7.4). The BAL fluid samples were then centrifuged for 7 min at 500 ×g, and the BAL cells were collected and resuspended in 1 mL of 0.9% NaCl solution for evaluation. The total cells number was determined using a hemocytometer. The cells were first smeared and then stained with Wright Giemsa Sure Stain for counting the total number of cells, macrophages, polymorphonuclear leukocytes (PMNs), and lymphocytes. The BAL levels of microalbumin and lactate dehydrogenase (LDH) were also measured using reagents obtained from Randox Laboratories Ltd. (London, UK) and Waco Pure Chemical Industries, Ltd. (Osaka, Japan), respectively. The level of inflammatory cytokines such as tumor necrosis factor-*α* (TNF-*α*), interleukin- (IL-) 1*β*, IL-18, granulocyte colony-stimulating factor (G-CSF), macrophage colony-stimulating factor (M-CSF), and vascular endothelial growth factor (VEGF) in the BAL fluid was measured using a Bio-Plex Rat 23-Plex assay (Bio-Rad Laboratories, Inc., Hercules, CA) according to the manufacturer's protocol. The level of tumor growth factor *β*1 (TGF-*β*1), matrix metallopeptidase-9 (MMP-9), and tissue inhibitor of metalloproteinase-1 (TIMP-1) was measured using Quantikine ELISA kit (R&D System, Minneapolis, MN) according to the manufacturer's instructions.

### 2.6. Lung Histopathology

At sacrifice, the left lungs were removed and fixed in a 10% formalin solution containing neutral PBS under 25 cm water pressure followed by embedding in paraffin and staining with hematoxylin and eosin. The stained lung tissue sections were examined under a light microscope for histopathological evaluation.

### 2.7. Statistical Analysis

The statistical analysis was performed using SPSS (Version 19) and the statistical evaluation performed using an analysis of variance (ANOVA) following multiple comparison tests using Duncan's method. The level of statistical significance was set at *P* < 0.05 and *P* < 0.01.

## 3. Results

### 3.1. Characteristics of Graphene Oxide

**Figure 1:**
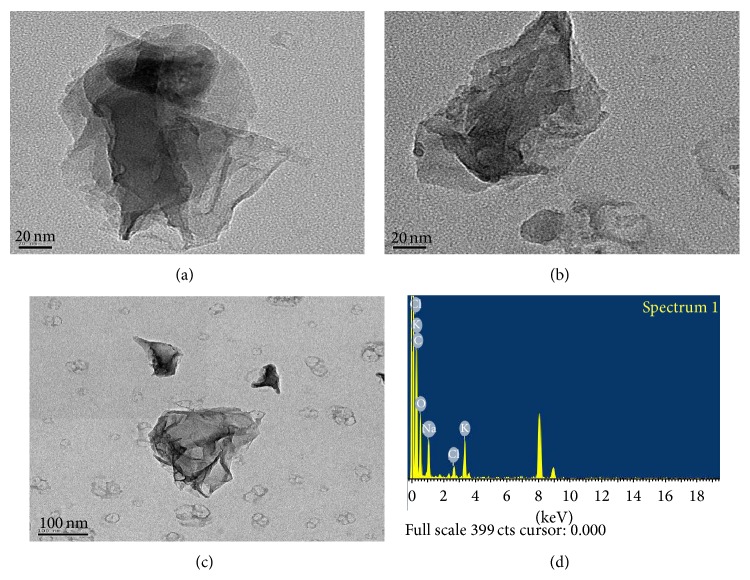
Analysis of graphene oxide nanomaterial using field emission-transmission electron microscope (FE-TEM, ×100,000) (a–c) and energy-dispersive X-ray spectrometer (EDS) (d).

**Figure 2:**
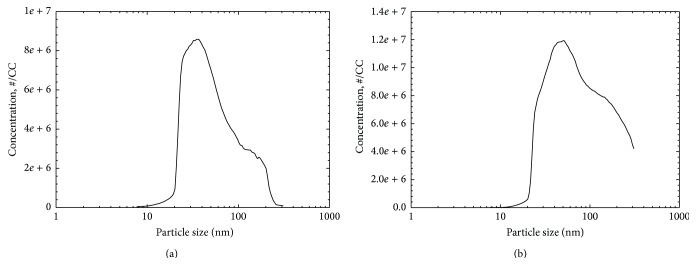
Particle size distribution of graphene oxide in low- (a) and high-concentration (b) chambers measured using scanning nanoparticle spectrometer (SNPS).

The field emission TEM (FE-TEM) analysis characterized the graphene oxide as a folded and stacked platelet structure (Figures 1(a)–1(c)). The TEM-EDS analysis in Figure 1(d) shows the presence of five elements (i.e., C, O, Na, Cl, and K), where the main elements were carbon and oxygen. Plus, Table 1 shows the atomic % of graphene oxide based on the EDS analysis: carbon (70.18%), oxygen (18.73%), sodium (5.38%), chlorine (1.42%), and potassium (4.29%). The XRD pattern for the natural graphite oxide revealed a sharp reflection at 2*θ* = 26.4°, corresponding to the interlayer spacing (*d* = 0.34 nm). The peak in the XRD pattern for the dried graphene oxide particles showed broadening, as well as a shift to a lower angle (2*θ* = 9.5°), indicating that the interlayers were 0.93 nm apart due to intercalation by the hydroxyl, carbonyl, and epoxide groups and moisture. The DLS measurement showed the average flake size of the graphene oxide in a solution. Plus, the equivalent hydrodynamic diameter of the graphene oxide was estimated using Stokes-Einstein equation to be 150–250 nm (Figure 2).

### 3.2. Monitoring Chamber and Graphene Oxide Distribution

The temperature and humidity were 23.10 ± 0.05°C and 32.90 ± 0.21%, respectively, for the low-concentration inhalation chamber and 25.25 ± 0.07°C and 34.40 ± 0.34%, respectively, for the high-concentration inhalation chamber. The graphene oxide exposure concentrations (mg/m^3^) measured based on the weight of the polycarbon filter before and after sampling were 0.46 ± 0.06 and 3.76 ± 0.24 for the low- and high-concentration groups, respectively (Table 2). The particles number (particles/cm^3^) in the chambers was measured directly using SNPS and was 3.33 × 10^6^ ± 2.95 × 10^5^ and 6.17 × 10^6^ ± 4.13 × 10^5^ for the low- and high-concentration groups of graphene oxide, respectively (Table 2). The diameter (nm) and surface area (nm^2^/cm^3^) of the graphene oxide were 50.6 ± 1.82 nm and 6.45 × 10^10^ ± 7.04 × 10^9^ nm^2^/cm^3^, respectively, for the low-concentration chamber and 72.9 ± 2.02 nm and 2.72 × 10^11^ ± 3.70 × 10^10^ nm^2^/cm^3^, respectively, for the high-concentration chamber (Table 2). The size distribution and number of graphene oxide particles in each chamber were also measured during the exposure period using SNPS (Figures 2(a) and 2(b)). The particle size ranged from 10 to 120 nm, with the highest peak at 35 nm and 50 nm for the low- and high-concentration chambers, respectively.

### 3.3. Animal Observation, Food Consumption, and Effect on Body and Organ Weights

No significant gross effects were observed during the exposure and recovery periods. Also, there were no significant differences in food intake between the control and graphene oxide-treated groups (see Supplement 1 in Supplementary Material available online at http://dx.doi.org/10.1155/2015/376756). No significant body weight changes were observed for the low- and high-concentration groups during the exposure and recovery periods (Supplement 2). The examination of the rat organs, including the testes, kidneys, spleen, liver, lungs, and brain, also revealed no significant clinical signs or organ weight changes during the observation period (Supplements 3–11).

### 3.4. Effects of Graphene Oxide Exposure in BAL Fluid

The levels of microalbumin and LDH were measured in the BAL fluid as indicators of bronchoalveolar mucosal permeability and lung cell damage, respectively. The results indicated that the single 6-hour nose-only inhalation exposure of the rats to graphene oxide did not significantly change the levels of microalbumin and LDH in the BAL fluid (Table 3). Plus, the results from counting the BAL cells (i.e., total cells, macrophages, PMNs, and lymphocytes) showed no significant alterations following the graphene oxide exposure at all the time points tested (Table 4). In order to determine the level of inflammation and damage in the lung, a total of nine toxicity parameters were measured in the cell-free BAL fluid. The results showed that limited numbers of parameters were increased with graphene oxide inhalation in the cell-free BAL fluid (Table 5). At day 1 after inhalation, MMP-9 was significantly increased in the high-concentration group compared to control and the low-concentration group. At day 7, IL-18 and TGF-*β*1 were significantly higher than those in control group. No significant alterations of toxicity parameters with graphene oxide exposure were observed at day 14.

### 3.5. Histopathology and High-Resolution Imaging

**Figure 3:**
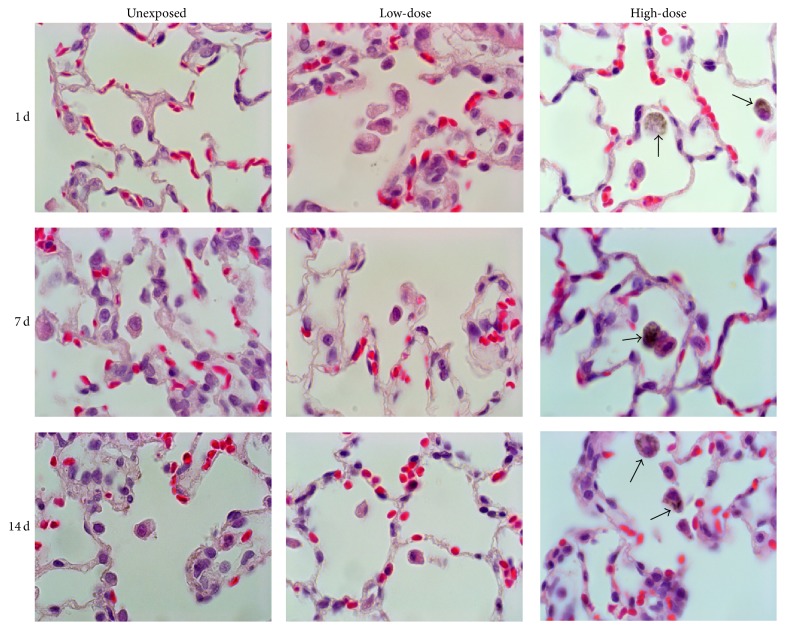
Lung histopathology after 6 h graphene oxide exposure, followed by 1, 7, or 14 days of recovery (×400). Black arrows indicate graphene oxide-loaded alveolar macrophages.

The histopathological examination of the rat lungs did not reveal any pathological changes in the low- and high-concentration groups after the graphene oxide exposure (Figure 3). However, alveolar macrophages with ingested graphene oxide were visualized in the high-concentration group during the recovery period, that is, 1 day, 7 days, and 14 days after exposure (Figure 3).

## 4. Discussion

Since graphene nanomaterials are experiencing rapid development with predicted widespread application, concerns about human exposure have also been recently increasing. However, safety information, particularly the inhalation toxicity of graphene-based nanomaterials, remains limited [11]. Therefore, this study investigated the toxic effects of graphene oxide nanomaterials, which were characterized as a folded and stacked platelet structure with a diameter of 150–250 nm. Graphene oxide is a chemically modified and highly oxidized form of graphene that consists of single-atom-thick carbon sheets [12]. Plus, graphene oxide nanomaterials usually have monolayer or multilayer flakes [11], and the graphene oxide used in this study was characterized with multilayer flakes. A nose-only inhalation technique with rats was used since inhalation is considered the major route of human exposure to graphene nanomaterials, particularly in occupational settings. This inhalation method has also been extensively used by the current authors for investigating nanomaterial toxicity [8, 13]. In the present study, male Sprague-Dawley rats were exposed to two different concentrations of graphene oxide (i.e., low-concentration group, 0.46 ± 0.06 mg/m^3^, and high-concentration group, 3.76 ± 0.24 mg/m^3^) in order to observe the causality and dose-dependent toxic responses in animals. Concentrations of graphene oxide were chosen based on preliminary data which presented dose-dependent toxic responses after 5-day repeated inhalation of graphene nanomaterial. Moreover, our concentration is more relevant to worker exposure levels compared to other previous study which used up to 10 mg/m^3^ [5]. For example, the worker exposure to multiwalled carbon nanotube (examined elemental carbon level) was found to be 5.5–7.3 *μ*g/m^3^ in the work area sampling [14].

Overall, no significant clinical changes due to graphene oxide exposure were observed during the 6-hour inhalation and 14-day recovery period, unlike other carbon nanomaterial toxicity studies such as MWCNTs and SWCNTs [8, 15, 16]. A slight decrease of body weight was observed for all the treatment groups, including the control, when comparing just before and after the exposure. However, this decrease of weight gain was likely due to exposure-related stress (i.e., the restriction in the nose-only chamber for 6 hours; see Supplement 2). In fact, a rat in the control group was found dead 1 day after exposure. The body weight gain remained normal during the rest of the recovery period until the termination of the study. The food intake was also similar in all the treatment groups, indicating that the graphene oxide inhalation did not affect the overall health of the animals (Supplement 1). Moreover, the gross findings for the rat organs, such as the testes, kidneys, spleen, liver, lungs, and brain, during the recovery time revealed no particular changes due to the graphene oxide exposure when compared to the control (Supplements 3–5). Neither the absolute nor the relative organ weights were significantly changed following the inhalation of graphene oxide (Supplements 6–11). Therefore, these results indicate that the 6-hour inhalation of graphene oxide was not enough to cause any adverse clinical signs at the concentrations selected in this study. Other past studies that used a nose-only inhalation method for rats also showed no adverse clinical signs due to graphene exposure [5], even with much higher concentrations of graphene nanomaterials (0.54 mg/m^3^, 3.05 mg/m^3^, and 10.1 mg/m^3^) and a longer period of inhalation (6 hours per day for 5 consecutive days) and similar concentration (0.68 and 3.86 3.05 mg/m^3^) and exposure period (6 hours per day for 5 consecutive days and 28 days after exposure) [10]. Thus, the present results and data from past studies suggest that graphene oxide is unlikely to cause significant clinical responses at relatively high concentrations in the air.

The present study also observed several toxicological indicators to determine the toxicity of graphene oxide in the exposed rats. However, the results showed limited level of toxicity in the lungs of the exposed rats. BAL fluid evaluation of nine inflammatory parameters (i.e., TNF-*α*, IL-1*β*, IL-18, G-CSF, M-CSF, VEGF, TGF-*β*1, MMP-9, and TIMP-1) demonstrated that only MMP-9 (day 1) and IL-18 and TGF-*β*1 (day 7) were significantly elevated due to exposure of graphene oxide at high concentrations. These elevated parameters returned to baseline level after 14-day recovery period. This temporary elevation of MMP-9, IL-18, and TGF-*β*1 may be due to phagocytosis of graphene oxide by alveolar macrophage and other types of lung cells [17–19]. Our histopathological examination observed graphene oxide loaded macrophages in the rat lungs at the high concentration. Further, the evaluation of markers in the cell-free BAL fluid demonstrated a lack of significant or dose-dependent changes in the levels of microalbumin and LDH. The BAL cell measurements also failed to verify any toxic effects due to the inhalation of graphene oxide. There was a nonsignificant increase of the total cells and macrophages numbers resulting from the high concentration of graphene oxide at day 1 and day 14 recovery time points. None of the treatment groups showed any PMNs and the lymphocyte numbers were negligible in the BAL fluid, indicating minimal inflammatory responses due to the graphene oxide exposure. This data is contrary to a recent study, where mouse lungs were exposed to 50 *μ*g of unoxidized graphene platelets using a pharyngeal aspiration technique and the results demonstrated significant increases in LDH and the total cells and PMNs numbers at 24 h after exposure [6]. However, it should be noted that the dose of 50 *μ*g graphene (approximately 2.5 mg/kg mouse) used in the previous study was significantly higher than the doses used in the present study, where the approximate deposited doses were 0.026 mg (0.087 mg/kg rat) for the high-concentration group and 0.003 mg (0.01 mg/kg) for the low-concentration group assuming a deposition rate of 10% and a minute ventilation of 0.19L/min in a 300 g rat [20]. This calculation is based on the following formula: daily deposited dose = graphene oxide concentration × minute volume × exposure duration × deposition efficiency. Therefore the deposited doses were as follows: low-concentration group, 0.46 mg/m^3^ × (0.19 L/min) × 6 h × 0.1 = 0.003 mg deposited a day (0.46/1000 × 0.19 × 360 min × 0.1 = 0.003); high-concentration group, 3.76 mg/m^3^ × (0.19 L/min) × 6 h × 0.1 = 0.026 mg deposited a day (3.76/1000 × 0.19 × 360 min × 0.1 = 0.026). Another previous study also showed markedly elevated damage parameters in the BAL fluid [5], where Wistar rats exposed to graphene inhalation (three concentrations: 0.54, 3.05, or 10.1 mg/m^3^ for 6 h/d, for 5 days with 7 or 28 days of recovery) showed an elevated number of total cells and lymphocytes at 10.01 mg/m^3^ and PMNs at 3.05 mg/m^3^. Yet, the particle concentrations were significantly higher than the concentrations used in the present study. Interestingly, the lowest concentration (0.54 mg/m^3^ 6 h/d, 6 days) in the previous study did not increase the lung toxicity parameters, and this concentration is similar to the high concentration (3.76 mg/m^3^, 6 h) used in the present study in terms of the approximate total deposited dose in the rat lungs. The previous study also found a dose-dependent increase in the LDH levels in the BAL fluid after exposure to graphene. Thus, when taken collectively, while the experimental designs differed significantly between the two studies, the dose would seem to be the major factor determining the toxicity of graphene.

The present study also observed dose-dependent particle-loaded macrophages in the lungs of the exposed rats. However, the graphene oxide-loaded macrophages were only found in the high-concentration group at the selected recovery time points (1 day, 7 days, and 14 days). Therefore, this data demonstrated that the exposure concentration was an important factor determining the amount of graphene oxide accumulated in the alveolar region of the rat lungs, and the graphene oxide used in this study persisted in the lungs up to 14 days after exposure. However, no other histopathological changes were found in the lungs of the exposed rats. Similarly, Ma-Hock et al. [5] also found a concentration-dependent increase of particle-loaded macrophages up to 28 days after exposure. Dose-dependent particle accumulation was also reported by Park et al. [4], where a higher accumulation of particles in mouse lung tissue was observed in the high-concentration group (5 mg/kg) compared to the low-concentration group (2.5 mg/kg) at 28 days after a single instillation. Therefore, similar to the parameters in the BAL fluid, the lung deposition was found to be dependent on the concentration of particles, and no lung toxicity was associated with the concentrations employed in the present study.

In conclusion, the results of this study suggest that the single inhalation of graphene oxide in male Sprague-Dawley rats exerted minimal toxic responses, indicating that the selected graphene oxide was relatively inert at the concentrations used. However, further studies with varying experimental settings are still needed to determine the toxicity of graphene oxide nanomaterials.

## Supplementary Material

Supplement 1: shows food intake of rats.
Supplement 2: shows body weight of rats before and after the exposure.
Supplement 3-5: shows gross findings of rats during the recovery period.
Supplement 6-11: shows absolute and relative organ weight of rats during the recovery period.

## Figures and Tables

**Table 1:** Analysis of graphene oxide.

Element	Weight (%)	Atomic (%)
C	56.79	70.18
O	20.19	18.73
Na	8.33	5.38
Cl	3.39	1.42
K	11.30	4.29

Total	100.00	100.00

**Table 2:** Distribution of graphene oxide in nose-only inhalation chamber.

Group	Number by SNPS (particles/cm^3^)	Diameter^†^ (nm)	Surface area (nm^2^/cm^3^)	Mass concentration (mg/m^3^)
Unexposed	9.99 × 10^2^ ± 8.10 × 10	NA	NA	0.01 ± 0.01
Low	3.33 × 10^6^ ± 2.95 × 10^5^	50.6 (1.82)	6.45 × 10^10^ ± 7.04 × 10^9^	0.46 ± 0.06
High	6.17 × 10^6^ ± 4.13 × 10^5^	72.9 (2.02)	2.72 × 10^11^ ± 3.70 × 10^10^	3.76 ± 0.24

Mean ± SE; ^†^GM (GSD).

**Table 3:** Concentrations of lung toxicity markers in cell-free BAL fluid.

Markers	Group	1 day	7 days	14 days
Microalbumin (mg/dL)	Control	1.13 ± 0.38	0.92 ± 0.12	1.34 ± 0.25
	Low	0.88 ± 0.11	0.76 ± 0.12	1.33 ± 0.12
	High	1.18 ± 0.15	1.03 ± 0.11	0.81 ± 0.10

Lactate dehydrogenase (U/L)	Control	53.50 ± 12.63	52.25 ± 6.96	46.00 ± 3.46
	Low	45.25 ± 6.36	39.25 ± 3.35	44.25 ± 5.63
	High	57.25 ± 13.23	42.00 ± 6.45	49.00 ± 18.70

Mean ± SE.

**Table 4:** Distribution of cells in BAL fluid.

Markers	Group	1 day	7 days	14 days
Total cell count (×10^6^/mL)	Unexposed	0.65 ± 0.09	0.83 ± 0.15	0.67 ± 0.28
	Low	0.64 ± 0.08	0.87 ± 0.14	0.57 ± 0.12
	High	0.80 ± 0.15	0.57 ± 0.19	0.93 ± 0.04

Macrophages (×10^6^/mL)	Unexposed	0.61 ± 0.08	0.80 ± 0.14	0.65 ± 0.26
	Low	0.62 ± 0.08	0.84 ± 0.13	0.55 ± 0.12
	High	0.78 ± 0.15	0.56 ± 0.18	0.91 ± 0.04

PMN (×10^6^/mL)	Unexposed	0.00 ± 0.00	0.00 ± 0.00	0.00 ± 0.00
	Low	0.00 ± 0.00	0.01 ± 0.00	0.00 ± 0.00
	High	0.00 ± 0.00	0.00 ± 0.00	0.00 ± 0.00

Lymphocytes (×10^6^/mL)	Unexposed	0.02 ± 0.01	0.02 ± 0.00	0.01 ± 0.01
	Low	0.01 ± 0.00	0.01 ± 0.00	0.00 ± 0.00
	High	0.01 ± 0.00	0.00 ± 0.00	0.01 ± 0.00

Mean ± SE.

**Table 5:** Concentrations of lung inflammatory and damage parameters in cell-free BAL fluid.

Markers	Group	1 day	7 days	14 days
TNF-*α* (pg/mL)	Control	38.15 ± 22.34	10.97 ± 8.31	14.52 ± 4.88
	Low	12.83 ± 7.88	34.27 ± 3.25	12.31 ± 3.82
	High	18.88 ± 6.02	34.62 ± 6.77	11.96 ± 4.97

IL-1*β* (pg/mL)	Control	12.23 ± 2.56	9.96 ± 1.63	16.69 ± 9.10
	Low	9.75 ± 1.24	9.93 ± 1.36	7.13 ± 0.93
	High	13.35 ± 2.79	18.84 ± 9.63	5.86 ± 2.06

IL-18 (pg/mL)	Control	127.44 ± 59.87	127.56 ± 65.09	151.64 ± 26.23
	Low	154.71 ± 76.44	323.24 ± 29.42	149.90 ± 38.64
	High	177.68 ± 59.95	361.68 ± 61.31^b^	126.06 ± 40.95

G-CSF (pg/mL)	Control	1.43 ± 0.36	1.52 ± 0.51	1.44 ± 0.20
	Low	1.33 ± 0.40	3.02 ± 0.40	1.56 ± 0.29
	High	1.76 ± 0.46	3.05 ± 0.54	1.24 ± 0.31

M-CSF (pg/mL)	Control	4.78 ± 0.70	4.61 ± 1.53	4.04 ± 0.60
	Low	4.04 ± 0.89	6.06 ± 0.41	4.05 ± 0.22
	High	6.38 ± 1.08	7.78 ± 1.58	2.83 ± 0.72

VEGF (pg/mL)	Control	311.18 ± 53.55	330.28 ± 49.59	348.50 ± 63.75
	Low	361.76 ± 40.87	379.78 ± 66.06	422.10 ± 82.85
	High	294.94 ± 49.84	464.30 ± 39.59	319.20 ± 16.98

TGF-*β*1 (pg/mL)	Control	7.64 ± 2.42	5.07 ± 1.63	11.26 ± 1.71
	Low	10.00 ± 2.40	9.93 ± 1.36	9.54 ± 2.56
	High	8.50 ± 0.82	14.79 ± 9.63^b^	12.77 ± 2.94

MMP-9 (ng/mL)	Control	18.92 ± 1.43	25.18 ± 7.14	24.79 ± 5.58
	Low	17.99 ± 0.57	13.49 ± 3.35	36.98 ± 18.02
	High	97.63 ± 27.91^a^	34.94 ± 6.91	24.54 ± 7.31

TIMP-1 (pg/mL)	Control	660.90 ± 91.26	686.85 ± 74.24	582.19 ± 78.56
	Low	688.04 ± 11.60	711.33 ± 52.30	580.34 ± 86.26
	High	1,037.93 ± 156.37	725.42 ± 115.16	663.89 ± 92.97

Mean ± SE.

^a^Significantly high versus control and low (*p* < 0.05).

^b^Significantly high versus control (*p* < 0.05).
