# Supplementary material for: Pulmonary Responses of Sprague-Dawley Rats in Single Inhalation Exposure to Graphene Oxide Nanomaterials
Source: Biomed Res Int. 2015 Jul 30;2015:376756. doi: 10.1155/2015/376756 (PMC4534591; doi:10.1155/2015/376756)
Supplement: Supplementary file 1 — Supplement 1: shows food intake of rats. Supplement 2: shows body weight of rats before and after the exposure. Supplement 3-5: shows gross findings of rats during the recovery period. Supplement 6-11: shows absolute and relative organ weight of rats during the recovery period. [file 376756.f1.pdf]

Supplements

Suppl. 1. Food consumption of rats (g/animal) during the recovery period

Group	Unexposed	Low	High
7-day recovery	29.35±0.33 (7)	28.8±0.32 (8)	29.22±0.76 (8)
14-day recovery	28.32±0.57 (3)	32.77±1.12 (4)	31.00±1.25x (4)

( ): number of animal

Suppl. 2. Body weight of rats (g/animal) before and after graphene oxide exposure

Group	Unexposed	Low	High
Before exposure	290.13±2.53 (12)	298.32±2.16 (12)	295.87±2.64 (12)
Necropsy (1-day recovery)	268.75±2.02 (4)	266.85±1.01 (4)	264.10±1.18 (4)
Necropsy (7-day recovery)	312.17±2.83 (4)	320.6±1.33 (4)	321.62±4.21 (4)
Necropsy (14-day recovery)	351.93±4.73 (3)	366.37±3.77 (4)	363.65±2.78 (4)

( ): number of animal

Suppl. 3. Gross findings of rats after 1 day following graphene oxide exposure

Organ	Observation	Unexposed	Low	High
TESTIS (LEFT)	Normal	4/4	4/4	4/4
TESTIS (RIGHT)	Normal	4/4	4/4	4/4
KIDNEY (LEFT)	Normal	4/4	4/4	4/4
KIDNEY (RIGHT)	Normal	4/4	4/4	4/4
SPLEEN	Normal	4/4	4/4	4/4
LIVER	Normal	4/4	4/4	4/4
LUNG (LEFT)	Normal	4/4	4/4	4/4
BRAIN	Normal	4/4	4/4	4/4

Suppl. 4. Gross findings of rats after 7 days following graphene oxide exposure

Organ	Observation	Unexposed	Low	High
TESTIS (LEFT)	Normal	4/4	4/4	4/4
TESTIS (RIGHT)	Normal	4/4	4/4	4/4
KIDNEY (LEFT)	Normal	4/4	4/4	4/4
KIDNEY (RIGHT)	Normal	4/4	4/4	4/4
SPLEEN	Normal	4/4	4/4	4/4
LIVER	Normal	4/4	4/4	4/4
LUNG (LEFT)	Normal	4/4	4/4	4/4
BRAIN	Normal	4/4	4/4	4/4

Suppl. 5. Gross findings of rats after 14 days following graphene oxide exposure

Organ	Observation	Unexposed	Low	High
TESTIS (LEFT)	Normal	3/4	4/4	4/4
TESTIS (RIGHT)	Normal	3/4	4/4	4/4
KIDNEY (LEFT)	Normal	3/4	4/4	4/4
KIDNEY (RIGHT)	Normal	3/4	4/4	4/4
SPLEEN	Normal	3/4	4/4	4/4
LIVER	Normal	3/4	4/4	4/4
LUNG (LEFT)	Normal	3/4	4/4	4/4
BRAIN	Normal	3/4	4/4	4/4

Suppl. 6. Absolute organ weight of rats (g/animal) after 1 day following graphene oxide exposure

Organ	Unexposed	Low	High
BODY WEIGHT	268.87±2.02 (4)	266.85±1.01 (4)	264.10±1.18(4)
TESTIS (LEFT)	1.40±0.05 (4)	1.37±0.04 (4)	1.24±0.03 (4)
TESTIS (RIGHT)	1.37±0.05 (4)	1.34±0.03 (4)	1.25±0.03 (4)
KIDNEY (LEFT)	1.08±0.01 (4)	1.09±0.02 (4)	1.07±0.02 (4)
KIDNEY (RIGHT)	1.07±0.00 (4)	1.08±0.02 (4)	1.01±0.02 (4)
SPLEEN	0.61±0.03 (4)	0.65±0.04 (4)	0.61±0.02 (4)
LIVER	8.31±0.04 (4)	8.93±0.18 (4)	8.31±0.15 (4)
LUNG (LEFT)	0.48±0.09 (4)	0.44±0.02 (4)	0.43±0.02 (4)
BRAIN	2.06±0.01 (4)	1.95±0.04 (4)	2.03±0.03 (4)

( ): number of animal

Suppl. 7. Absolute organ weight of rats (g/animal) after 7 days following graphene oxide exposure

Organ	Unexposed	Low	High
BODY WEIGHT	312.17±2.83 (4)	320.60±1.33 (4)	321.62±4.21 (4)
TESTIS (LEFT)	1.36±0.01 (4)	1.43±0.01 (4)	1.39±0.02 (4)
TESTIS (RIGHT)	1.32±0.01 (4)	1.42±0.00 (4)	1.36±0.03 (4)
KIDNEY (LEFT)	1.21±0.03 (4)	1.30±0.03 (4)	1.28±0.03 (4)
KIDNEY (RIGHT)	1.20±0.01 (4)	1.24±0.02 (4)	1.24±0.05 (4)
SPLEEN	0.72±0.04 (4)	0.67±0.04 (4)	0.67±0.04 (4)
LIVER	9.91±0.19 (4)	10.62±0.22 (4)	10.15±0.39 (4)
LUNG (LEFT)	0.43±0.01 (4)	0.42±0.02 (4)	0.41±0.01 (4)
BRAIN	2.05±0.06 (4)	2.04±0.07 (4)	1.97±0.02 (4)

( ): number of animal

Suppl. 8. Absolute organ weight of rats (g/animal) after 14 days following graphene oxide exposure

Organ	Unexposed	Low	High
BODY WEIGHT	351.93±4.73 (3)	366.37±3.77 (4)	363.65±2.78 (4)
TESTIS (LEFT)	1.75±0.08 (3)	1.85±0.07 (4)	1.55±0.04 (4)
TESTIS (RIGHT)	1.68±0.02 (3)	1.77±0.07 (4)	1.51±0.06 (4)
KIDNEY (LEFT)	1.53±0.03 (3)	1.53±0.05 (4)	1.46±0.04 (4)
KIDNEY (RIGHT)	1.53±0.03 (3)	1.52±0.06 (4)	1.47±0.05 (4)
SPLEEN	0.84±0.07 (3)	0.76±0.06 (4)	0.78±0.07 (4)
LIVER	11.80±0.42 (3)	11.38±0.50 (4)	11.76±0.15 (4)
LUNG (LEFT)	0.48±0.02 (3)	0.48±0.01 (4)	0.42±0.01 (4)
BRAIN	2.33±0.08 (3)	2.24±0.06 (4)	2.10±0.07 (4)

Suppl. 9. Relative organ weight of rats (g/animal) after 1 day following graphene oxide exposure

Organ	Unexposed	Low	High
BODY WEIGHT	268.87±2.02 (4)	266.85±1.01 (4)	264.10±1.18 (4)
TESTIS (LEFT)	0.52±0.02 (4)	0.51±0.01 (4)	0.46±0.01 (4)
TESTIS (RIGHT)	0.51±0.02 (4)	0.50±0.01 (4)	0.47±0.01 (4)
KIDNEY (LEFT)	0.40±0.00 (4)	0.40±0.00 (4)	0.40±0.01 (4)
KIDNEY (RIGHT)	0.39±0.00 (4)	0.40±0.01 (4)	0.38±0.01 (4)
SPLEEN	0.23±0.01 (4)	0.24±0.01 (4)	0.23±0.01 (4)
LIVER	3.09±0.02 (4)	3.34±0.06 (4)	3.14±0.06 (4)
LUNG (LEFT)	0.17±0.03 (4)	0.16±0.01 (4)	0.16±0.00 (4)
BRAIN	0.76±0.01 (4)	0.73±0.02 (4)	0.76±0.01 (4)

( ): number of animal

Suppl. 10. Relative organ weight of rats (g/animal) after 7 days following graphene oxide exposure

Organ	Unexposed	Low	High
BODY WEIGHT	312.17±2.83 (4)	320.60±1.33 (4)	321.62±4.21 (4)
TESTIS (LEFT)	0.43±0.00 (4)	0.44±0.00 (4)	0.43±0.01 (4)
TESTIS (RIGHT)	0.42±0.00 (4)	0.44±0.00 (4)	0.42±0.01 (4)
KIDNEY (LEFT)	0.39±0.01 (4)	0.40±0.00 (4)	0.39±0.00 (4))
KIDNEY (RIGHT)	0.38±0.00 (4)	0.38±0.00 (4	0.38±0.01 (4)
SPLEEN	0.23±0.01 (4)	0.21±0.01 (4)	0.21±0.01 (4)
LIVER	3.17±0.07 (4)	3.31±0.06 (4)	3.15±0.01 (4)
LUNG (LEFT)	0.13±0.00 (4)	0.13±0.00 (4)	0.12±0.00 (4)

BRAIN	0.65±0.02 (4)	0.63±0.02 (4)	0.61±0.01 (4)

( ): number of animal

Suppl. 11. Relative organ weight of rats (g/animal) after 14 days following graphene oxide exposure

Organ	Unexposed	Low	High
BODY WEIGHT	351.93±4.73 (3)	366.37±0.377 (4)	363.65±2.78 (4)
TESTIS (LEFT)	0.49±0.02 (3)	0.50±0.02 (4)	0.42±0.00 (4)
TESTIS (RIGHT)	0.47±0.00 (3)	0.48±0.02 (4)	0.41±0.01 (4)
KIDNEY (LEFT)	0.43±0.01 (3)	0.41±0.01 (4)	0.40±0.01 (4)
KIDNEY (RIGHT)	0.43±0.01 (3)	0.41±0.01 (4)	0.40±0.01 (4)
SPLEEN	0.24±0.02 (3)	0.20±0.01 (4)	0.21±0.02 (4)
LIVER	3.35±0.12 (3)	3.10±0.12 (4)	3.23±0.04 (4)
LUNG (LEFT)	0.13±0.00 (3)	0.13±0.00 (4)	0.11±0.00 (4)
BRAIN	0.66±0.01 (3)	0.61±0.01 (4)	0.58±0.02 (4)

( ): number of animal
